# Two-Stage Conversion of High Free Fatty Acid *Jatropha curcas* Oil to Biodiesel Using Brønsted Acidic Ionic Liquid and KOH as Catalysts

**DOI:** 10.1155/2014/180983

**Published:** 2014-03-30

**Authors:** Subrata Das, Ashim Jyoti Thakur, Dhanapati Deka

**Affiliations:** ^1^Department of Energy, Tezpur University, Tezpur, Assam 784028, India; ^2^Department of Chemical Sciences, Tezpur University, Tezpur, Assam 784028, India

## Abstract

Biodiesel was produced from high free fatty acid (FFA) *Jatropha curcas* oil (JCO) by two-stage process in which esterification was performed by Brønsted acidic ionic liquid 1-(1-butylsulfonic)-3-methylimidazolium chloride ([BSMIM]Cl) followed by KOH catalyzed transesterification. Maximum FFA conversion of 93.9% was achieved and it reduced from 8.15 wt% to 0.49 wt% under the optimum reaction conditions of methanol oil molar ratio 12 : 1 and 10 wt% of ionic liquid catalyst at 70°C in 6 h. The ionic liquid catalyst was reusable up to four times of consecutive runs under the optimum reaction conditions. At the second stage, the esterified JCO was transesterified by using 1.3 wt% KOH and methanol oil molar ratio of 6 : 1 in 20 min at 64°C. The yield of the final biodiesel was found to be 98.6% as analyzed by NMR spectroscopy. Chemical composition of the final biodiesel was also determined by GC-MS analysis.

## 1. Introduction

Biodiesel is chemically fatty acid alkyl esters of long-chain fatty acids derived from renewable sources such as vegetable oils and animal fats through the esterification and transesterification reactions of FFAs and triglycerides, respectively [1]. In transesterification, vegetable oil is allowed to be reacted with methanol in the presence of a catalyst which results into biodiesel and glycerol. Currently, more than 95% of the world biodiesel is produced from edible oils which are easily available on large scale from the agricultural industry [2]. However, continuous and large-scale production of biodiesel from edible oils has recently been of great concern because they compete with food materials, the food versus fuel dispute [3]. Also these expensive edible vegetable oils make the biodiesel production process costly as the feedstocks only account up to 75% of the total production cost [4]. In this context, the utilization of low cost nonedible oils as feedstock is very significant in developing countries [5]. However, the biodiesel production from nonedible oils by utilizing conventional homogenous alkaline catalysts like NaOH and KOH is not possible due to the chances of saponification owing to its high free fatty acid (FFA) content (>1% w/w) [4]. Alternatively acid catalysts like sulphuric acid which are insensitive to the FFA content could catalyze the esterification and transesterification reaction simultaneously. But this approach is also unsuitable because of its slow reaction rate (4000 times slower as compared to alkali catalyzed transesterification) [6]. So none of the processes are suitable and convenient for crude oils. To address this issue a two-stage process is developed where, in the first stage, acid catalysts (such as H_2_SO_4_) are utilized to lower the FFA content below 1 wt% followed by second stage alkaline catalyzed transesterification [7]. Again this traditional homogenous acid catalyst (sulphuric acid) suffers from some serious disadvantages such as nonreusability of the catalyst, problem of waste disposal, and requirement of costly equipment owing to the corrosive nature of the acid [8]. Recently, biodiesel production by two-stage process with regard to acidic ionic liquid catalysts and deep eutectic solvents has been reported in scientific literature [9–12]. Ionic liquids are adopted as catalysts because they are considered as designer solvents and also have some green characteristics such as extremely low vapour pressure, nonflammability, reusability, and high thermal stability which makes the process clean [13, 14].

**Scheme 1:**
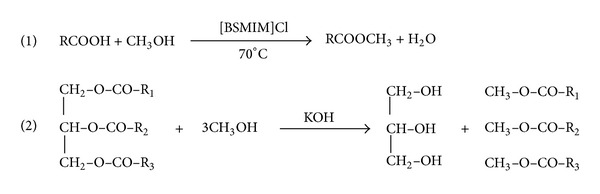
Transesterification of triglycerides with alcohol (usually with methanol).

In present investigation, the aim is to report a new acidic ionic liquid for the pretreatment or esterification of crude JCO and to study the efficiency of the catalyst in esterification with regard to catalyst dosage and other reaction parameters such as methanol oil molar ratio, reaction temperature, and also reaction time and reusability of it. The particular ionic liquid can also be developed to a new and novel series of ionic liquid catalysts that can catalyze not only the esterification of FFAs of crude oil but also the esterification and transesterification reactions of crude oils for the production of biodiesel efficiently and simultaneously. Additionally the synthetic procedure of the ionic liquid is very simple and easy since no additional solvent is necessary and can be carried out simply by stirring at room temperature and is also atom efficient since no byproducts were generated during the synthesis. The reactions scheme adopted in this study is shown in Scheme 1.

## 2. Materials and Methods

### 2.1. Materials

Local dried* Jatropha* seeds were collected from the Kaliabor Nursery, Kaliabor, Assam, India. 1-Methylimidazole (99%), 1,4-butane sultone (≥99%), and 2-propanol (anhydrous, 99.5%) were procured from Sigma Aldrich, India. Methanol (≥99%, GC grade), hexane (fraction from petroleum), HCl (35% for analysis), and KOH (analytical grade) were purchased from Merck India Limited. All the chemicals were used as received without any further purification.

### 2.2. Instruments

IR spectra were recorded in KBr pallets on a Nicolet (Impact 410) FT-IR spectrophotometer. ^1^H and ^13^C NMR spectra were recorded in a 400 MHz NMR spectrophotometer (JEOL, JNM ECS) using tetramethylsilane (TMS) as the internal standard and coupling constants are expressed in Hertz. Elemental analyses were carried out in a Perkin-Elmer CHN analyzer (2400 series II).

The fatty acid composition was determined by GC-MS (Perkin Elmer Clarus 600) equipped with TCD detector and elite wax-5MS capillary column (30 m × 0.25 mm × 0.25 *μ*m). The GC oven was kept at 50°C for 2 min, heated up to 250°C at the rate of 10°C/min increase in temperature, and kept at this temperature for 5 min and the total analytical time taken was 27 min. The carrier gas used for the purpose was helium and the flow rate was maintained at 1 mL/min. The UV-visible spectra were recorded on Shimadzu UV-1700 spectrophotometer. The solutions of the ionic liquid (10 m mol/L), H_2_SO_4_ (10 m mol/L), and 4-nitroaniline (10 m mol/L) were made in distilled water and their UV-vis spectra were carried out in the scale of 220–600 nm at room temperature. ESI-MS spectrum was obtained on Agilent 6410 Triple Quad MS-MS instrument.

The thermal analysis of the ionic liquid was measured by Pyris Diamond TG analyzer (Perkin Elmer) with a heating rate of 10°C/min from room temperature to 500°C. A high purity N_2_ gas (99.99%) was used as a carrier gas at a flow rate of 10 mL min^−1^. In the experiment, a sample weighing approximately 28 mg was used.

### 2.3. Extraction and Purification of JCO

The extraction of JCO was performed with the help of Soxhlet apparatus by using hexane (boiling point 65–70°C) as solvent according to the AOAC method 2003.06. The oil was separated from solvent by means of rotary vacuum evaporator. The collected JCO was then filtered to remove all the solid impurities that may be present in the oil followed by heating at 100°C for 10 min to remove the remaining moisture [15].

### 2.4. Preparation of Ionic Liquid

#### 2.4.1. Preparation of Zwitterion

**Scheme 2:**
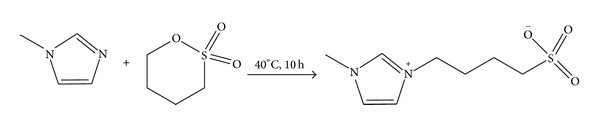


The zwitterion was prepared according to the procedure reported in [16] and is illustrated in Scheme 2. In a 100 mL round-bottom flask, equal moles of 1-methylimidazole and 1,4-butane sultone were added and then the mixture was stirred for 10 h at 40°C. The zwitterion formed appeared as white solid and was dried in vacuum after being washed repeatedly with ether to remove unreacted reactants and impurities, if any. Quantitative yield of the product was obtained which was then characterized as follows: ^1^H NMR (400 MHz, D_2_O, TMS): *δ* ppm 1.61–1.68 (m, 2H), 1.89–1.96 (m, 2H), 2.82–2.87 (m, 2H), 3.79 (s, 3H), 4.13 (t, *J* = 7.9 Hz, 2H), 7.34-7.35 (m, 1H), 7.40-7.41 (m, 1H), 8.64 (s, 1H). ^13^CNMR (100 MHz, D_2_O, TMS): *δ* ppm 21.00, 28.17, 35.73, 48.99, 50.14, 122.25, 123.74, 136.06. IR (KBr): *γ* (3423.13, 3154.95, 3102.97, 1641.31, 1572.49, 1184.02, 1041.36, 738.34, 606.81, 531.11). Elemental analysis: calc. for C_8_H_14_N_2_O_3_S: C 44.02, H 6.46, N 12.83. Found: C 43.43, H 6.57, N 12.39%.

#### 2.4.2. Preparation of 1-(1-Butylsulfonic)-3-methylimidazolium Chloride ([BSMIM]Cl)

**Scheme 3:**
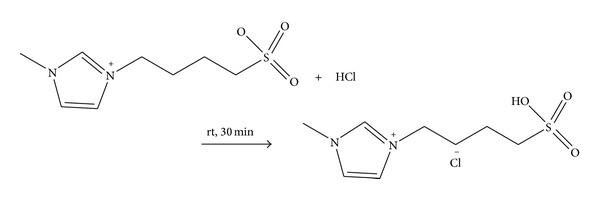


The ionic liquid was prepared according to reaction Scheme 3. In this process a stoichiometric amount of 35% w/w concentrated hydrochloric acid was added to the zwitterion, stirred for 30 min at room temperature to form the viscous ionic liquid and then kept in vacuum desiccator prior to use. ^1^H NMR (400 MHz, methanol-D_3_, TMS): *δ* ppm 1.76–1.84 (m, 2H), 2.02–2.10 (m, 2H), 2.84 (t, *J* = 7.9 Hz, 2H), 3.95 (s, 3H), 3.98 (s, 1H), 4.27 (t, *J* = 7.9 Hz, 2H), 7.60 (m, 1H), 7.69 (m, 1H), 9.01 (s, 1H). ^13^CNMR (100 MHz, methanol-D_3_, TMS): *δ* ppm 22.93, 30.06, 36.66, 50.38, 51.67, 123.82, 125.12, 138.22. IR (KBr): *γ* (3420.69, 3159.18, 2955.67, 1639.73, 1574.35, 1176.56, 1040.00, 606.61, 536.67). Elemental analysis: calc. for C_8_H_15_ClN_2_O_3_S: C 37.72, H 5.94, N 11.00. Found: C 37.53, H 5.97, N 10.79%. ESI-MS positive mass peaks (m/z) ([BSMIM]^+^):219.0, 437.1,655.1,873. 2. The thermal decomposition point of ionic liquid was 320°C.

### 2.5. Two-Step Biodiesel Production Process

#### 2.5.1. Ionic Liquid Catalyzed Esterification of JCO

The experiments were carried out in laboratory scale. Esterification was carried out by adding 20 gm of JCO, appropriate methanol, and [BSMIM]Cl ionic liquid as catalyst in a three-neck round-bottom flask which was attached to a chilled water cooled condenser and placed on a hot plate magnetic stirrer. The reaction was prolonged for a specific amount of time at the desired temperature with vigorous stirring. After the reaction, the mixture was then centrifuged and a biphasic mixture was obtained. The esterified JCO forms the upper layer which was separated by simple decantation, while the lower layer forms the mixture of ionic liquid, excess methanol, and water generated from the reaction. The lower layer of the biphasic mixture was then subjected to vacuum distillation to separate the water and excess methanol from the ionic liquid. The recovered ionic liquid was washed with diethyl ether, dried under vacuum, and reused for the next run. The esterified JCO was heated to remove moisture, dried over anhydrous Na_2_SO_4_, and then further subjected to alkali catalyzed transesterification. Conversion data were calculated based on acid value determination by using the following equation [17]:

(1) $$\begin{array}{c} \text{Conversion}\;\left(\%\right)=\left(\frac{a_{i}-a_{t}}{a_{i}}\right)\times 100, \end{array}$$

where *a*_*i*_ is initial acid value of the mixture and *a*_*t*_ is the acid value at any “*t*” time.

#### 2.5.2. Alkali Catalyzed Transesterification of JCO

The esterification was followed by transesterification of the esterified JCO by using KOH as catalyst according to the procedure reported earlier [18]. The reaction conditions were similar to those used during the esterification. In the reactor, the esterified JCO was heated and then mixed with the preheated solution of 1.3 wt% (based on the weight of JCO) KOH and methanol. A 6 : 1 molar ratio of methanol to JCO was taken and the mixture was stirred vigorously at 64°C for 20 min. After the reaction, the components of the reaction mixture were allowed to be separated from each other in a separating funnel under gravity separation where two layers were formed. The upper layer was the desired biodiesel which was separated by decantation and then washed with warm distilled water to make it free from impurities such as soap, unreacted methanol, and residual KOH. The washed biodiesel was then heated to remove moisture and dried over anhydrous Na_2_SO_4_ followed by analysis by Nuclear Magnetic Resonance (NMR) spectroscopy and GC-MS. The conversion of FAMEs was determined by the peak areas of the signals at 3.65 (protons of the methyl ester moiety) and at 2.31 (protons of the carbonyl methylene groups). The conversion was calculated by using the following simple equation [19]:

(2) $$\begin{array}{c} C=\frac{2A_{\text{ME}}}{3A_{\alpha \text{-}{\text{CH}}_{2}}}\times 100, \end{array}$$

where *C* is the conversion of JCO to the corresponding methyl ester, *A*_ME_ is the integration value of the protons of the methyl esters (the strong singlet peak), and *A*_*α*-CH_2__ is the integration value of the carbonyl methylene protons.

## 3. Results and Discussion

### 3.1. Feedstock Analysis

The physiochemical properties of JCO as determined according to the standard methods and the fatty acid methyl ester composition of JCO as determined by GC-MS chromatogram are given in Table 1. Five major peaks were observed in the chromatogram which was identified by comparing with the earlier reported publications [7] and the profiles from the NIST and Wiley GC-MS libraries. These five main components were identified as cis-9-hexadecenoic acid (0.65%), hexadecanoic acid (16.34%), cis-9, cis-12-octadecenoic acid (27.53%), cis-9-octadecenoic acid (47.30%), and octadecanoic acid (8.17%).

### 3.2. Determination of *H*_0_ Value of the Brønsted Acidic IL

The Brønsted acidity of the ionic liquid was determined from the determination of the Hammett acidity functions by using the following formula [20]:

(3) $$\begin{array}{c} H_{0}=\text{pK}{\left(\text{I}\right)}_{\text{aq}}+\log\left(\frac{\left[\text{I}\right]}{\left[\text{I}{\text{H}}^{+}\right]}\right), \end{array}$$

where pK(I)_aq_ is the pK_*a*_ value of the indicator and [I] and [IH^+^] are, respectively, the molar concentrations of the unprotonated and protonated forms of the indicator, which can usually be determined by UV-visible spectroscopy.

**Figure 1:**
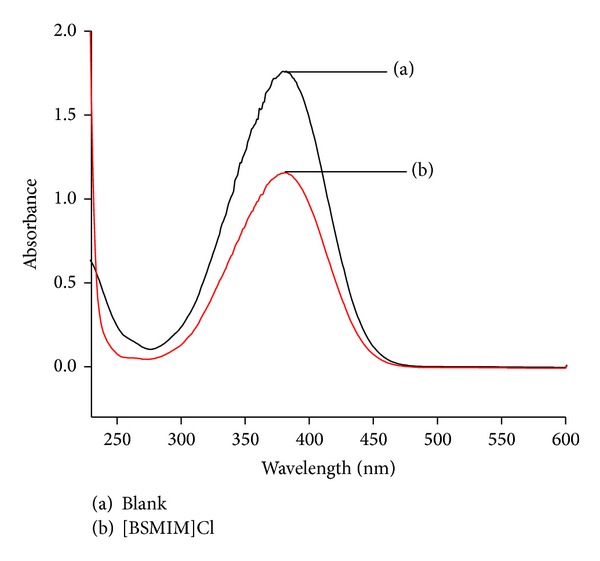
Absorption spectra of 4-nitroaniline for Brønsted acids in distilled H_2_O.

4-Nitroaniline (Hammett content is 0.99; concentration is 10 mmol/L) was used as an indicator in water [21] and the results are shown in Figure 1. The maximum absorbance of the unprotonated form of the indicator was observed at 382 nm in water as shown in the Figure 1, which is the same as the observed earlier [21]. The absorbance maxima decrease with the addition of acid. [I]/[IH^+^] ratio could be determined by measuring the absorbance differences and then the Hammett function (*H*_0_) is calculated (see Table 2).

### 3.3. [BSMIM]Cl-Catalyzed Esterification Reaction of JCO 

#### 3.3.1. Effect of the Catalyst Loading

**Figure 2:**
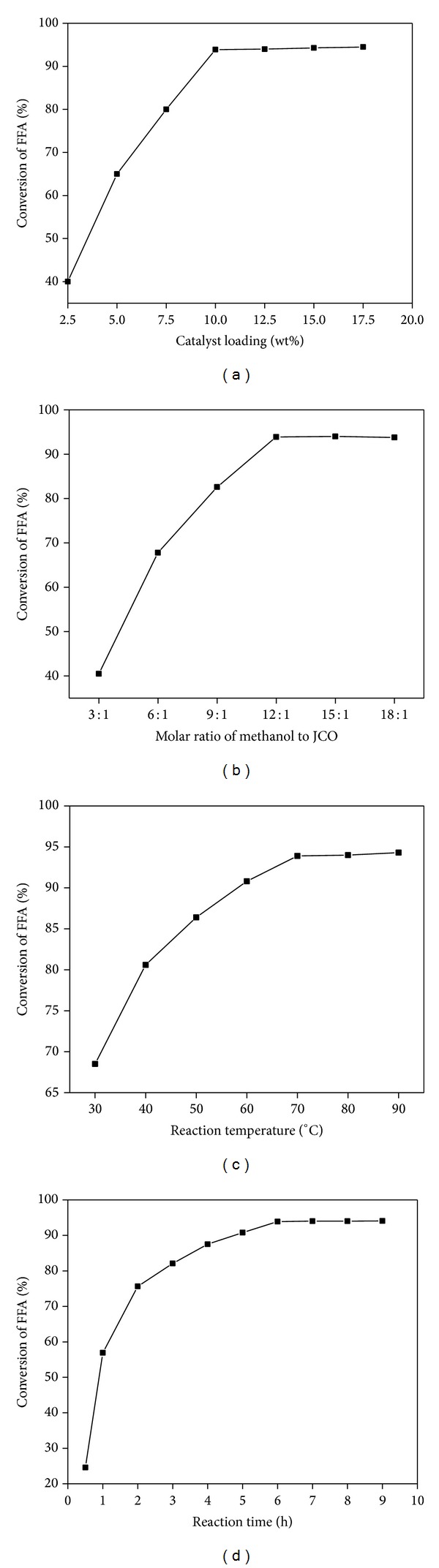
(a) Effect of the loading of [BSMIM]Cl on FFA conversion. Reaction conditions:* n*(methanol) :* n*(JCO) = 12 : 1; 70°C; 6 h. (b) Effect of molar ratio of methanol to JCO on FFA conversion. Reaction conditions:* n*(JCO) :* n*([BSMIM]Cl) : = 1 : 0.32; 70°C; 6 h. (c) Effect of the reaction temperature on FFA conversion. Reaction conditions:* n*(methanol) :* n*(JCO) :* n*([BSMIM]Cl) = 12 : 1: 0.32; 6 h. (d) Effect of the reaction time on FFA conversion. Reaction conditions:* n*(methanol) :* n*(JCO) :* n*([BSMIM]Cl) = 12 : 1 : 0.32; 70°C.

The catalyst loading is one of the most important factors that influence the esterification reaction and it is shown in Figure 2(a). The catalyst loading was varied in the range of 2.5–17.5 wt% (based on the weight of JCO) and FFA conversion was determined. From the figure it could be seen that FFA conversion was low at lower catalyst loading and it increased with the increment of catalyst loading. The maximum conversion of 93.9% was observed with 10 wt% of catalyst loading and further increase did not increase the conversion so much. Therefore the optimum catalyst loading was chosen as 10 wt%.

#### 3.3.2. Effect of Molar Ratio of Methanol to JCO

In esterification, generally an excess of methanol is necessary for the complete conversion of FFA. Additionally, since the ionic liquid catalyst was soluble in methanol, an excess of methanol should increase the reaction rate due to the enhancement of contact area between the reactants. Keeping this view in mind, the molar ratio of methanol to JCO was varied from 3 : 1 to 18 : 1 and the results are shown in the Figure 2(b). The results indicate that the FFA conversion increases with the increase of molar ratio of methanol to oil and reaches its highest value of 93.9% with 12 : 1 molar ratio, beyond which a slight increase in conversion is obtained. Therefore, the optimum methanol to JCO oil ratio was taken as 12 : 1.

#### 3.3.3. Effect of Reaction Temperature

The reaction temperature has an important role in esterification reaction. Generally reaction rate is enhanced with the rise in temperature, the effect of which on FFA conversion is shown in Figure 2(c). The figure reveals that under similar reaction conditions, higher FFA conversion is achieved at elevated temperature than at low temperature which may be due to the increment of the reaction rate at higher temperature. At low reaction temperature of 30°C, FFA conversion of 68.5% was achieved and it reached 93.9% at 70°C. The conversion did not improve much with further increase in temperature up to 90°C which means that the reaction attends the equilibrium at 70°C. Therefore, the suitable reaction temperature was chosen as 70°C.

#### 3.3.4. Effect of Reaction Time

The influence of reaction time is the last but not the least very important factor in esterification of FFA. The influence of reaction is presented in Figure 2(d). A reaction usually approaches equilibrium with increase in reaction time and then at a certain interval of time it attends the equilibrium. So it is very important to find out the time interval at which the esterification reaction reaches the equilibrium. It is evident from the figure that the FFA conversion increases with the increase in reaction time. The sample of the reaction mixture was extracted from the reactor at 1 h interval of time and the FFA conversion was determined. It was observed that the FFA conversion increases sharply at the initial stage of the reaction and then increases slowly up to 6 h of time. The conversion of 93.9% was obtained in 6 h and after that the conversion remained nearly constant. The result confirms that the catalyst is sufficiently active in esterification of FFA and reaches equilibrium only in 6 h at 70°C. So, 6 h was taken as the optimum reaction time.

### 3.4. Reusability Study of [BSMIM]Cl in Esterification of FFA of JCO

**Figure 3:**
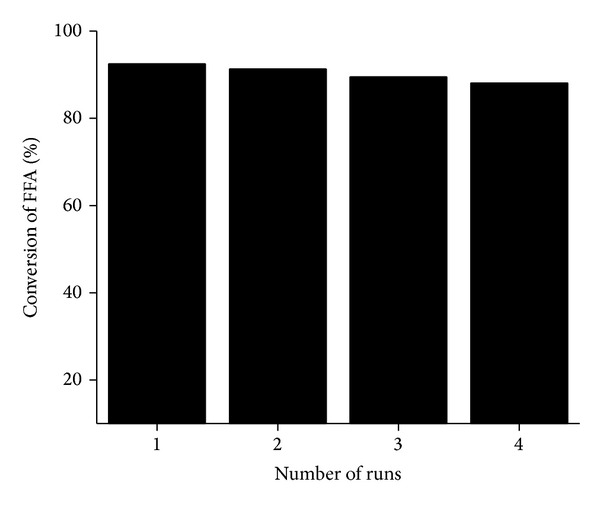
Reusability study of [BSMIM]Cl.

**Figure 4:**
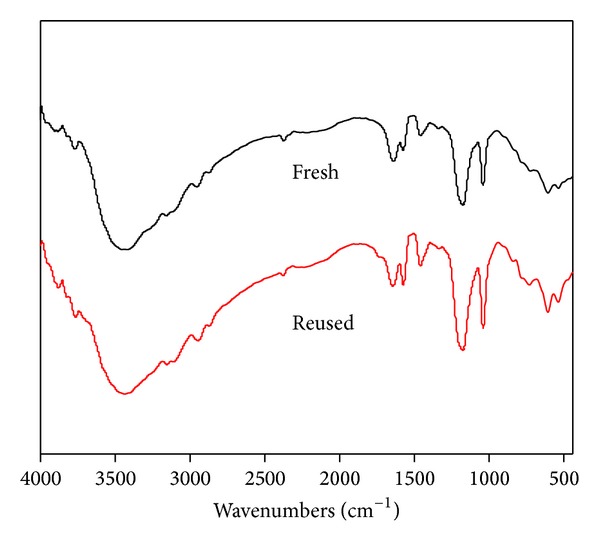
FT-IR spectrum comparison of the fresh catalyst and the four-time used catalyst.

The catalytic activity of the recycled ionic liquid (Table 3 and Figure 3) was investigated by performing esterification reactions for four consecutive runs under the optimum reaction conditions as determined earlier. The [BSMIM]Cl ionic liquid could be reused at least four times with any significant decrease in catalytic activity. The slight decrease in catalytic activity of ionic liquid may be due to gradual loss of ionic liquid in each run. The FT-IR spectrum comparison of fresh and the recovered catalyst after four runs depicted that there is no significant change in the ionic liquid structure (Figure 4). The reusability study reveals that up to four runs the esterified JCO had FFA content less than 1 wt% and hence could be used for the second step alkali catalyzed transesterification reaction for the production of biodiesel. These results indicate that the catalyst was very stable under these reaction conditions.

### 3.5. Alkali Catalyzed Transesterification

The esterified JCO used for the alkali catalyzed transesterification has 0.49 wt% FFA. This low FFA content of the oil makes it suitable for the alkali catalyzed transesterification process. Therefore, transesterification was carried out with 1.3 wt% KOH as catalyst, 6 : 1 molar ratio of methanol to JCO, and reaction temperature of 64°C for 20 min, and FAME conversion of 98.6% was obtained. Therefore the ester content in the final biodiesel meets the limit of 96.5% of European Biodiesel Standards (EN 14214).

### 3.6. Analysis of Final Biodiesel

#### 3.6.1. NMR Spectroscopic Analysis

**Figure 5:**
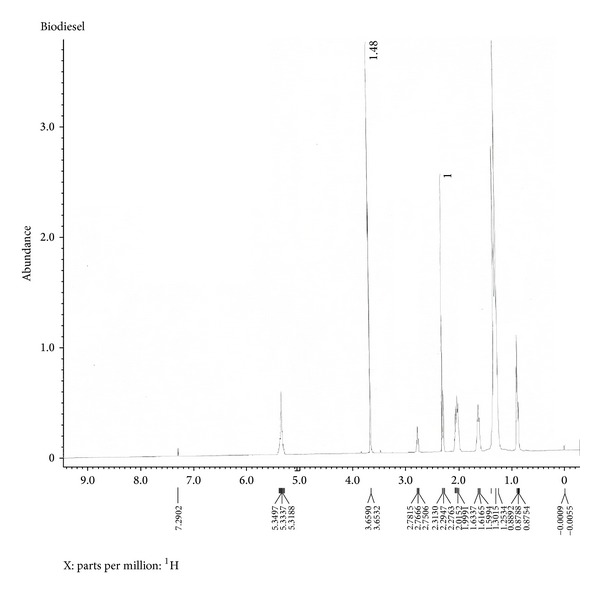
^1^H NMR spectra of final biodiesel.

**Figure 6:**
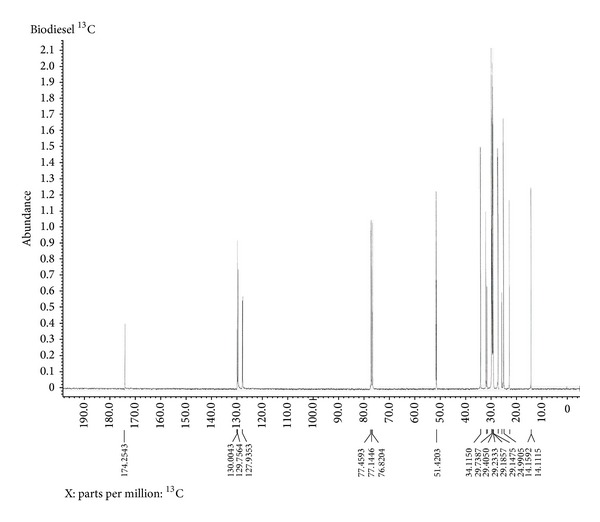
^13^C NMR spectra of final biodiesel.

The final biodiesel was analyzed by NMR spectroscopy and is shown in Figures 5 and 6. The peaks at 3.65 ppm of methoxy(–OCH_3_) group and 2.31 ppm of *α*-CH_2_ protons in ^1^H NMR spectra and at 174.25 ppm of ester carbonyl (–COO–) and 51.42 ppm of C–O in ^13^C NMR spectra confirm the presence of methyl esters in the biodiesel sample.

#### 3.6.2. GC/MS Analysis

**Figure 7:**
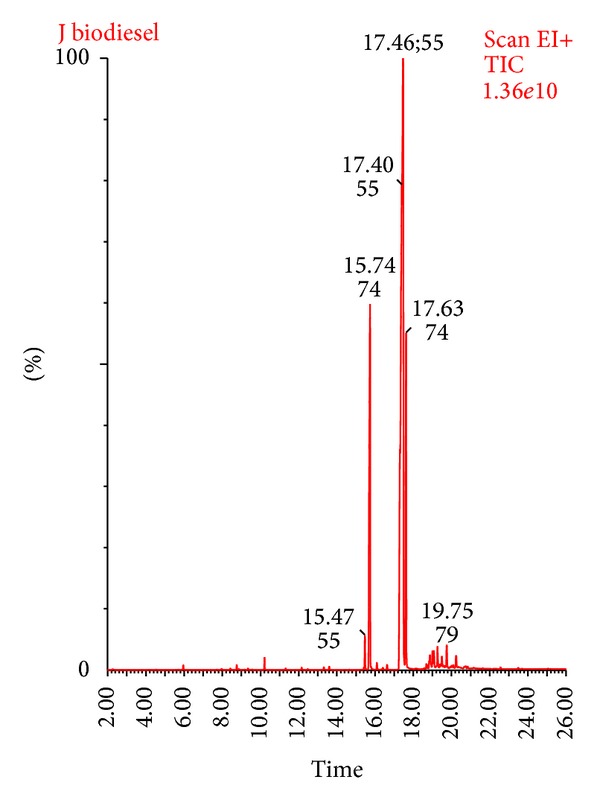
GC-MS of final biodiesel.

The chemical composition of the final biodiesel was determined by GC-MS analysis. Total ion chromatogram (TIC) (Figure 7) shows that there are five major peaks of five fatty acid methyl esters which are identified from NIST and Wiley GC/MS libraries and are listed in Table 4.

## 4. Conclusion

Maximum FFA conversion of 93.9% was obtained at mild reaction temperature of  70°C and the esterified JCO produced could be separated simply by centrifugation. The ionic liquid was also reusable up to four times with a slight loss in catalytic activity. Therefore, the utilization of [BSMIM]Cl in pretreatment process paves the way for the application of low cost feedstocks such as JCO for the continuous biodiesel production and lowers the overall cost in this process.

## Supplementary Material

Response of the author: The supplementary material contains the characterizations of both the zwitterions and the ionic liquid. The zwitterions was characterized by IR ,^1^H and ^13^C NMR spectroscopy and the ionic liquid was characterized by IR, ^1^H and ^13^C NMR spectroscopy, ESI-MS and TGA

## Figures and Tables

**Table 1:** Fatty acid composition and important properties of JCO.

Sl. number	Properties	JCO
	Fatty acid composition (wt%)	

1	Palmitic (16:0)	16.34
	Palmitoleic (16:1)	0.65
	Stearic (18:0)	8.17
	Oleic (18:1)	47.30
	Linoleic (18:2)	27.53
	Total saturated	24.51
	Total unsaturated	75.48

2	FFA (wt%)	8.156

3	Acid value (mgKOH/gm)	16.232

4	Saponification value (mgKOH/gm)	210.0

5	Average molecular weight (g/mol)	871

**Table 2:** *H*_0_ values of the ionic liquid in water at room temperature^a^.

Entry	Brønsted acid	*A*_max⁡_	[I] (%)	[IH^+^] (%)	*H*_0_
1	Blank	1.762	100.0	0	—
2	[BSMIM]Cl	1.154	65.494	34.506	1.268

^a^Concentration: 10 m mol/L; indicator: 4-nitroaniline.

**Table 3:** Reusability study of ionic liquid^b^.

Run	Conversion of FFA (%)	Acid value	FFA (wt%)
1	92.5	1.217	0.61
2	91.3	1.412	0.70
3	89.5	1.704	0.85
4	88.1	1.932	0.97

^b^Reaction conditions: *n*(methanol) : JCO : *n*([BSMIM]Cl) = 12 : 1 : 0.32; 70°C; 6 h.

**Table 4:** Fatty acid composition of final biodiesel.

FAMEs composition	Structure	Fatty acids (wt%)
Methyl palmitate	C16:0	16.08
Methyl palmitoleate	C16:1	0.77
Methyl stearate	C18:0	11.04
Methyl cleate	C18:1	46.50
Methyl linoleate	C18:2	25.61
