# Supplementary material for: Two-Stage Conversion of High Free Fatty Acid Jatropha curcas Oil to Biodiesel Using Brønsted Acidic Ionic Liquid and KOH as Catalysts
Source: ScientificWorldJournal. 2014 Mar 30;2014:180983. doi: 10.1155/2014/180983 (PMC3988968; doi:10.1155/2014/180983)

Two stage conversions of high free fatty acid *Jatropha curcas* oil to biodiesel using Brønsted acidic ionic liquid and KOH as catalysts.

Subrata Das*[1] , Ashim Jyoti Thakur[2], Dhanapati Deka[1].

[1]Dept of Energy, Tezpur University, Tezpur- 784028, Assam, India.

[2]Dept of Chemical Sciences, Tezpur University, Tezpur- 784028, Assam, India.

[*] Corresponding author.sdas@tezu.ernet.in. [1]Dept of Energy, Tezpur University, Tezpur- 784028, Assam, India.

Fig S1. ^{1}HNMR spectrum of Zwitterion.

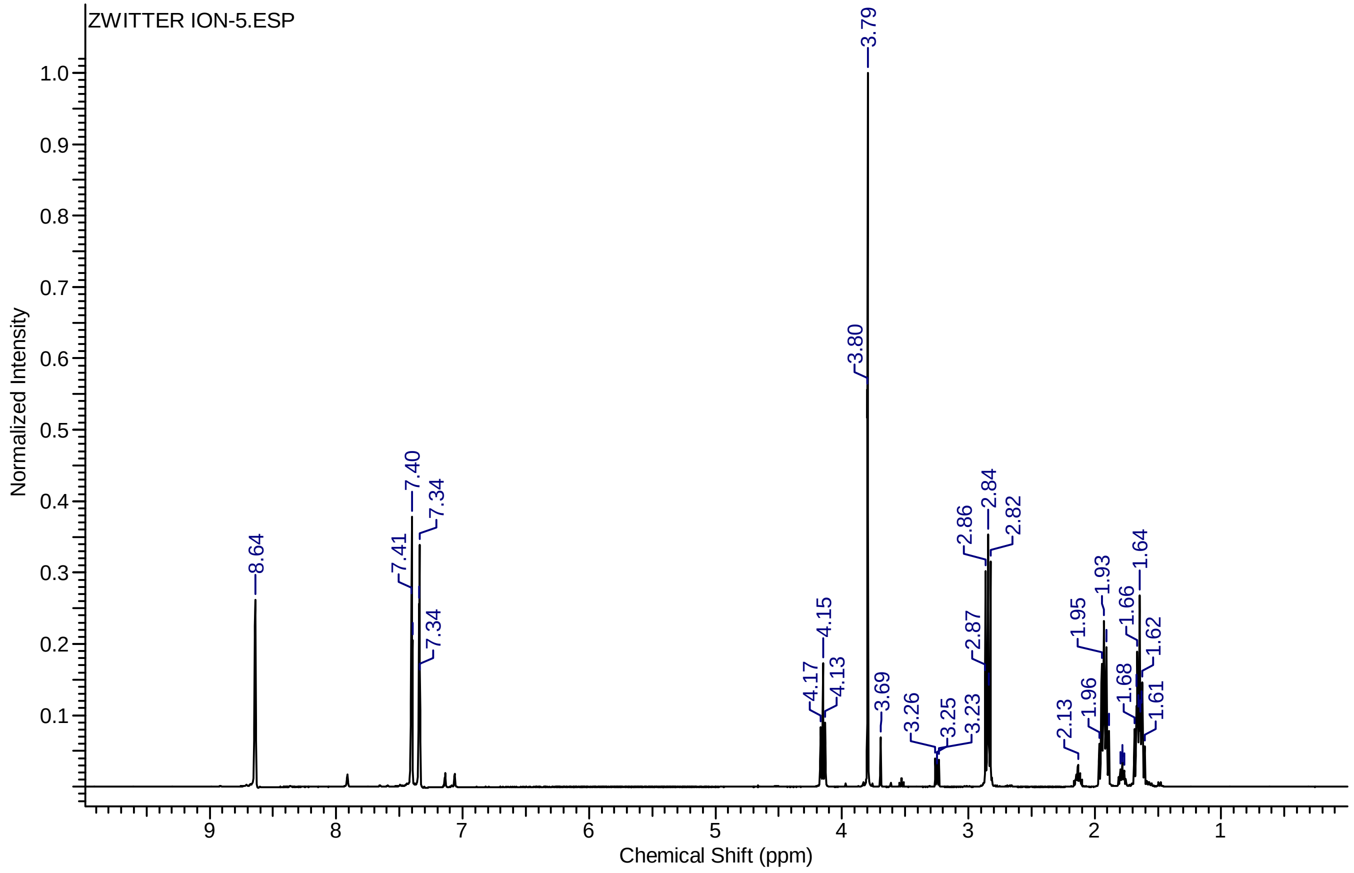

Fig. S2. ^{13}CNMR of Zwitterion

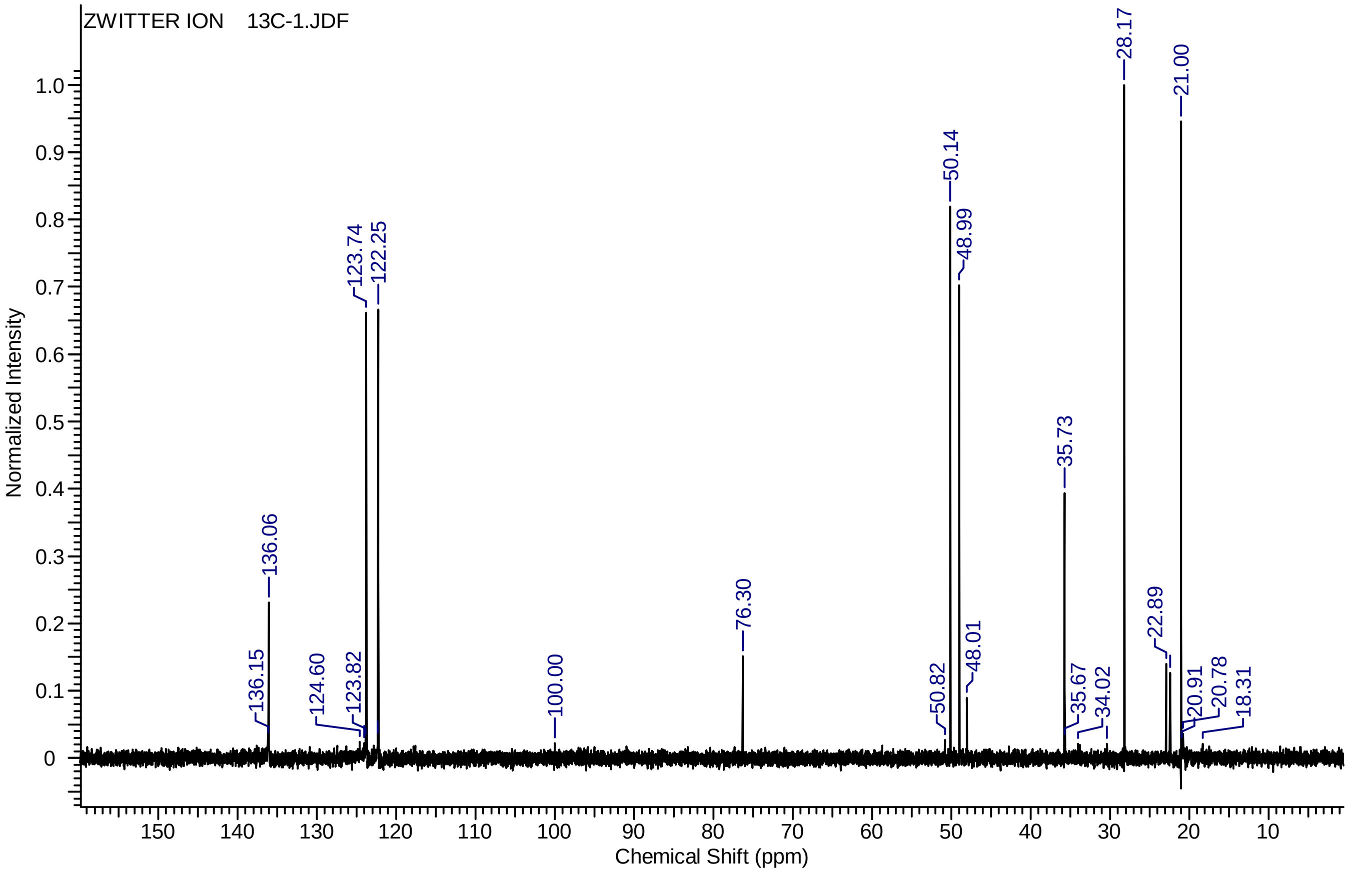

Fig. S3. IR of Zwitterion

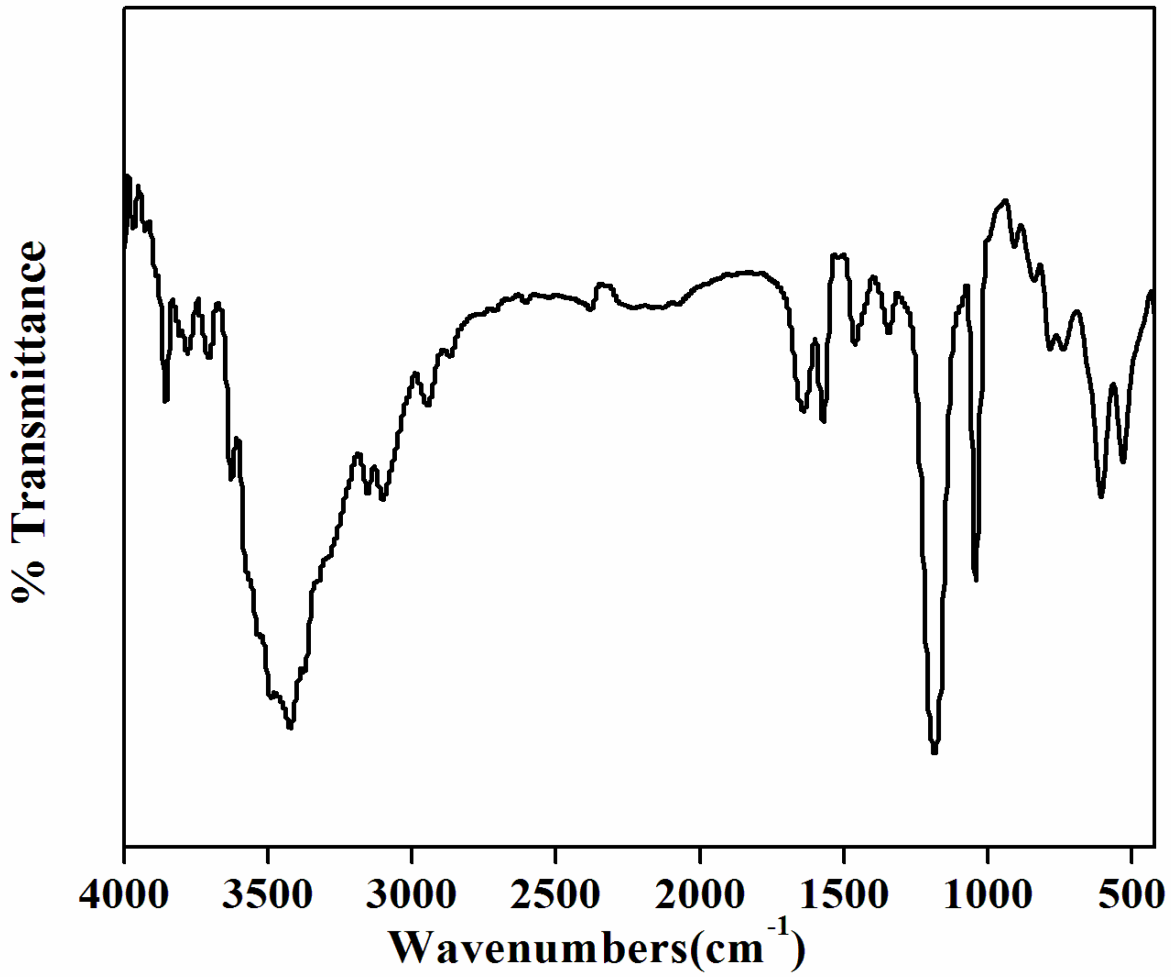

Fig S4 ^{1}HNMR spectrum of [BSMIM]Cl

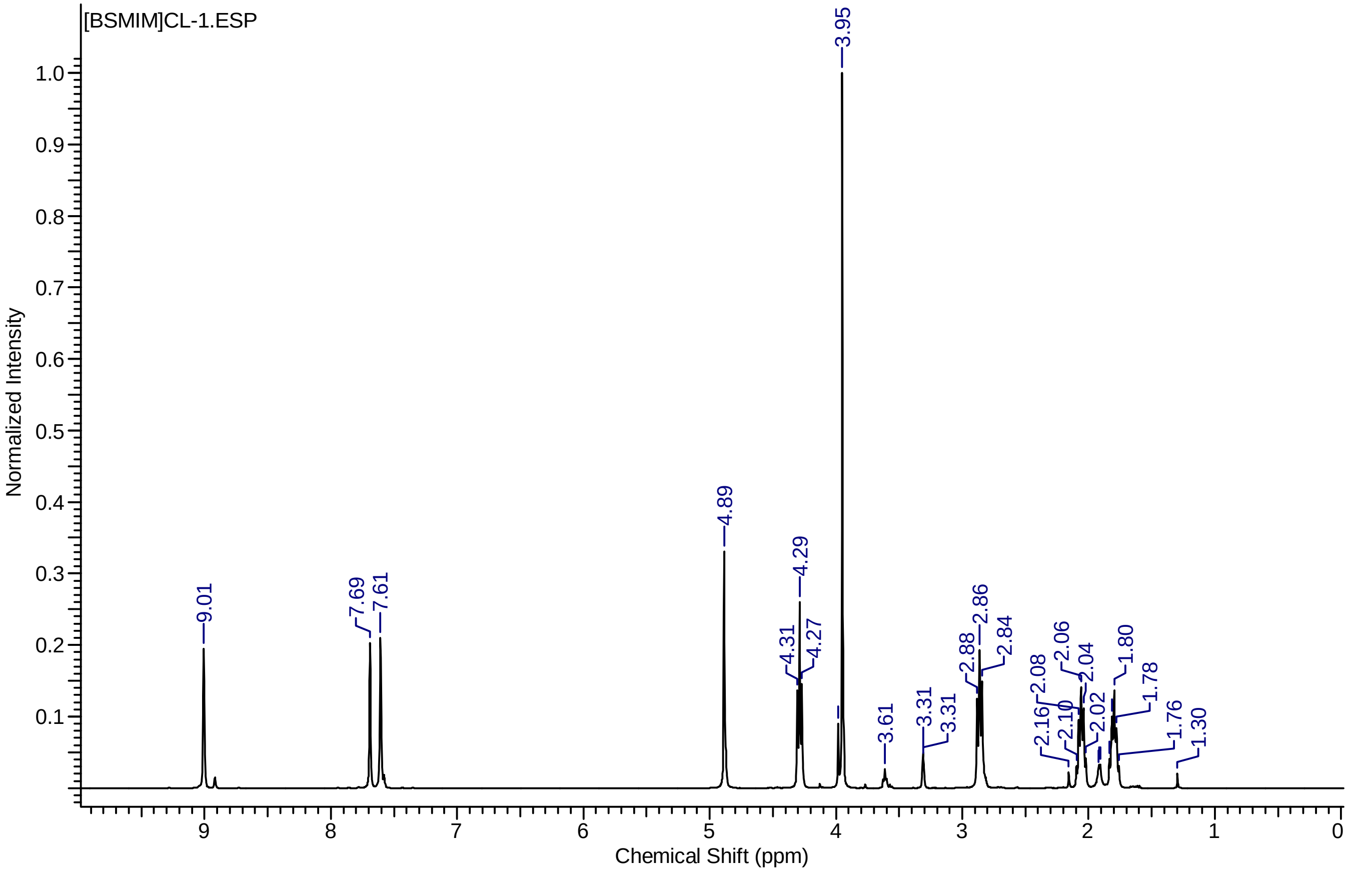

Fig. S5 ^{13}CNMR of [BSMIM]Cl

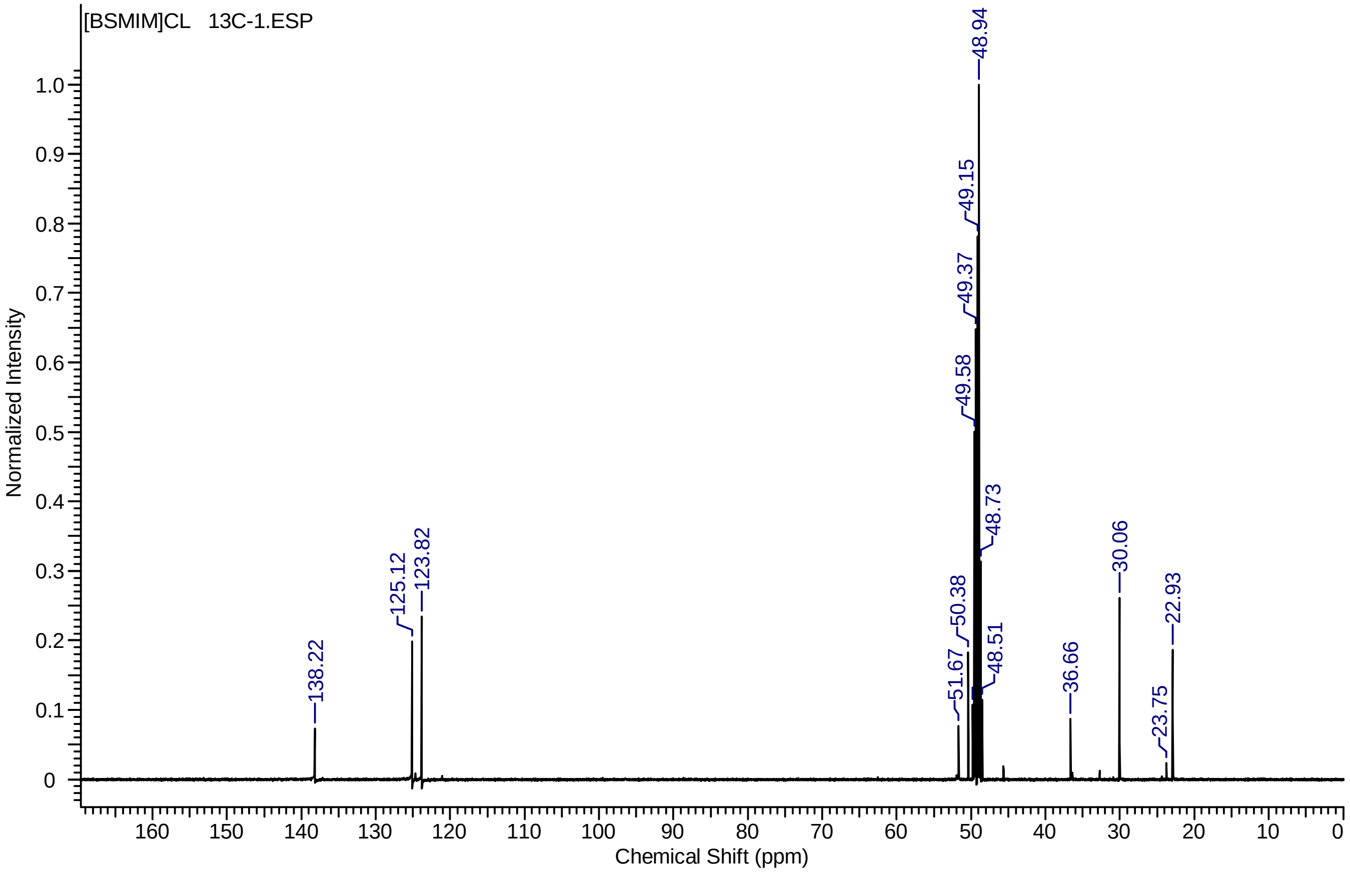

Fig. S6 IR of [BSMIM]Cl

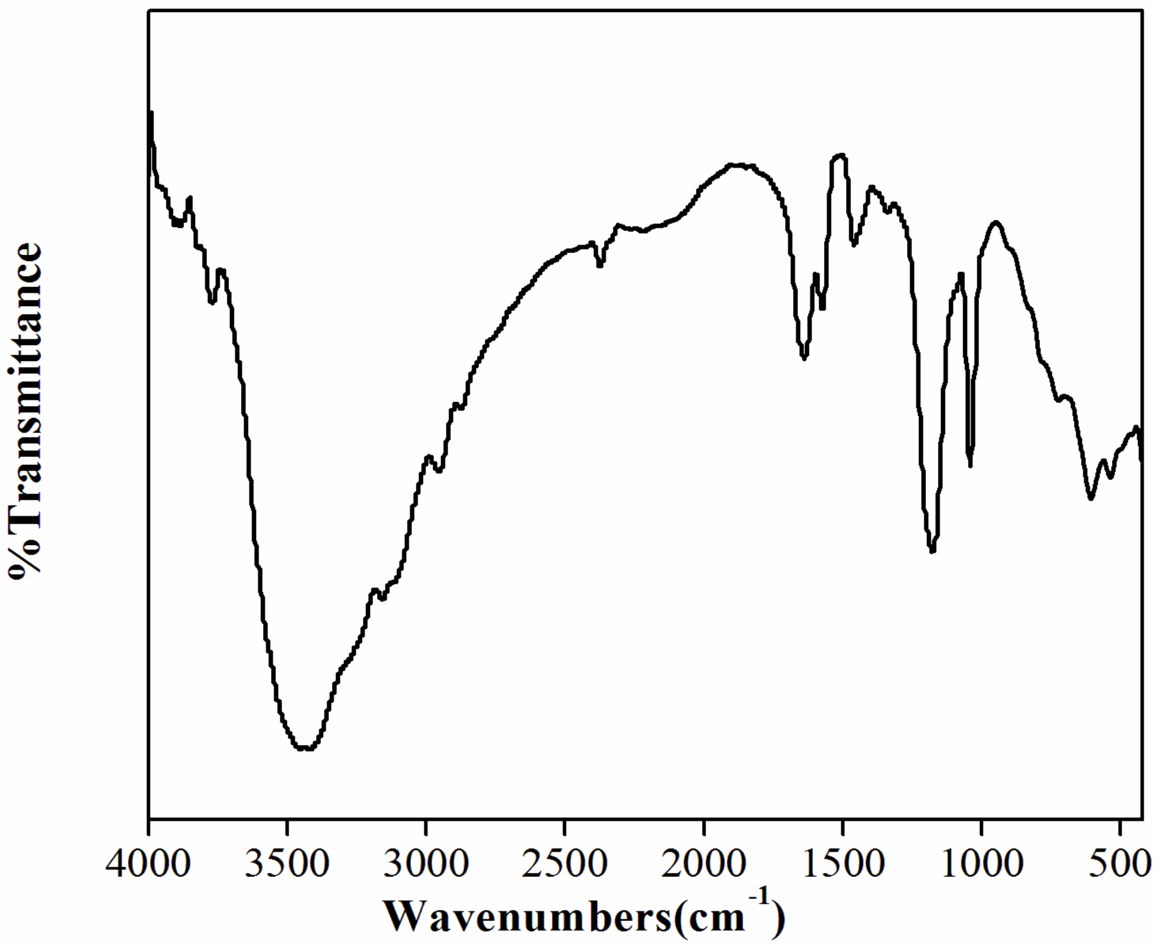

FigS7: ESI-MS of [BSMIM]Cl

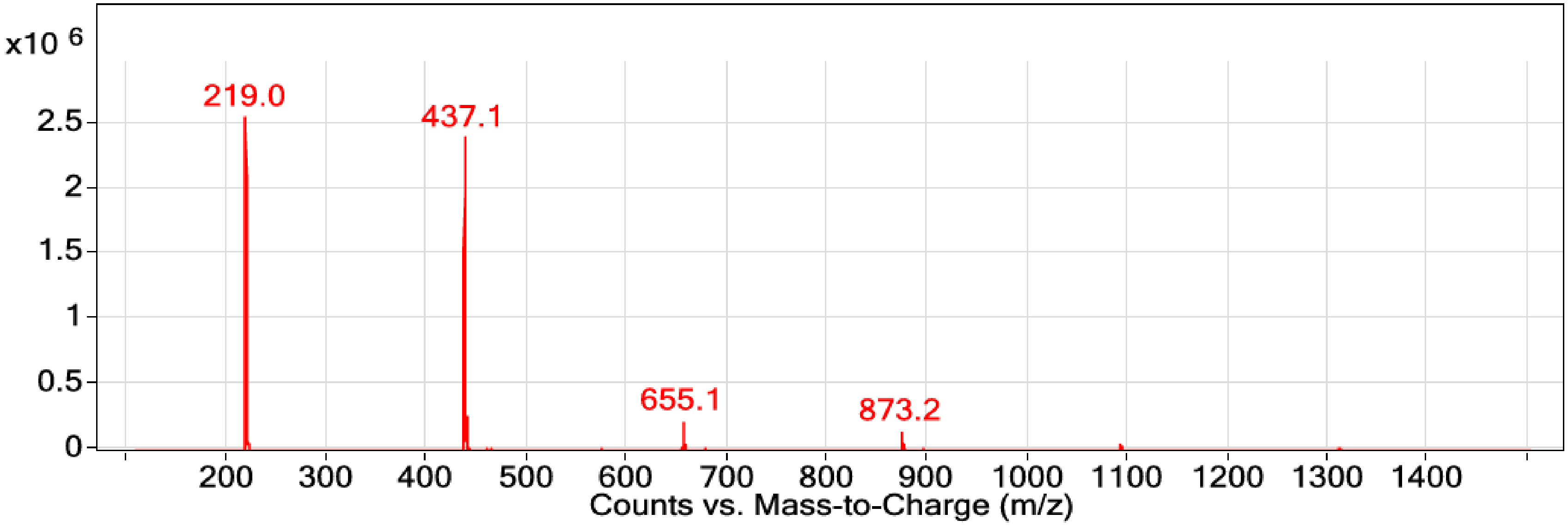

FigS10: TGA of [BSMIM]Cl

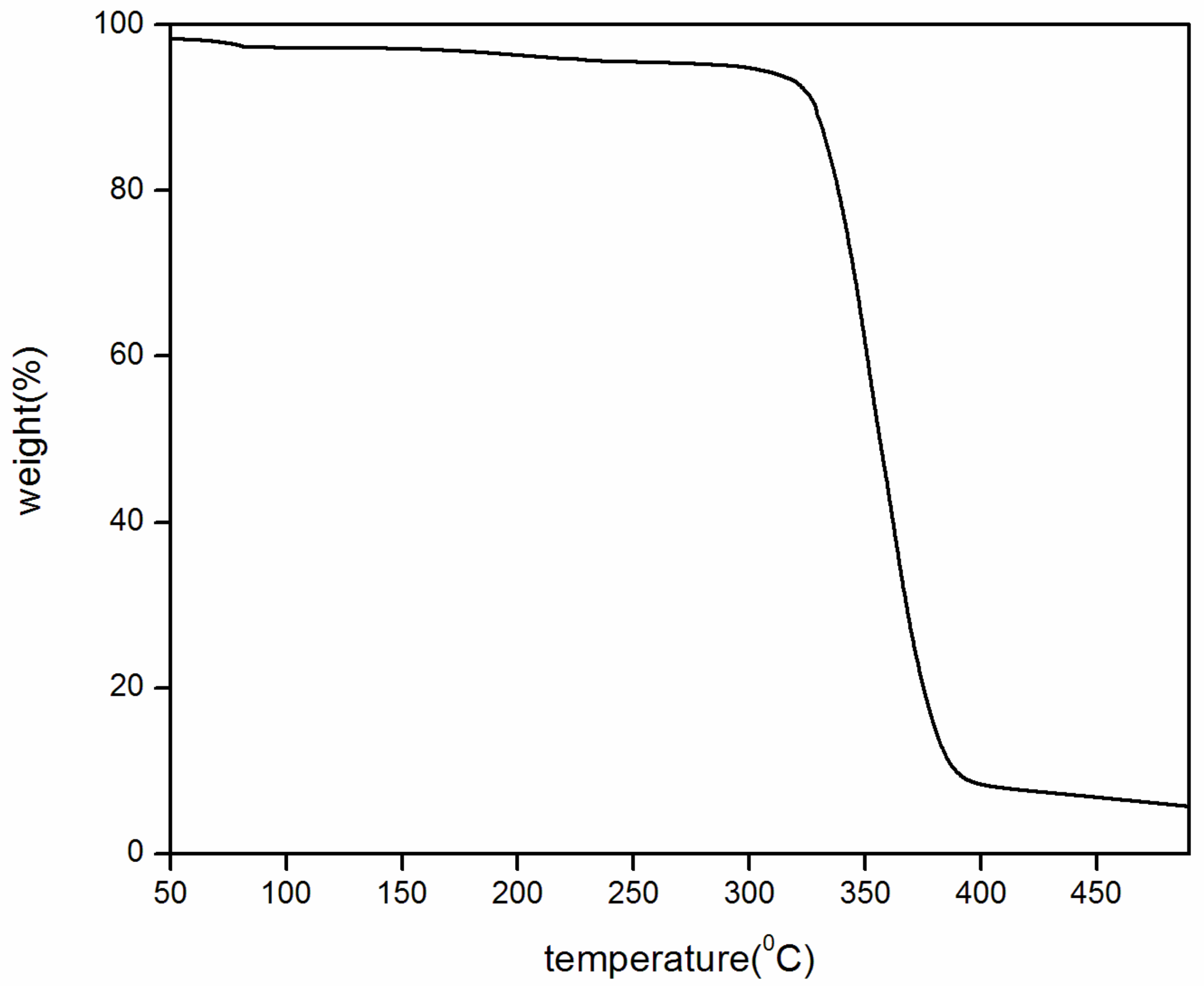

Supplement: Supplementary file 1 — Response of the author: The supplementary material contains the characterizations of both the zwitterions and the ionic liquid. The zwitterions was characterized by IR ,1H and 13C NMR spectroscopy and the ionic liquid was characterized by IR, 1H and 13C NMR spectroscopy, ESI-MS and TGA [file 180983.f1.pdf]
